# Ultrasound-Guided Regional Anesthesia for Repeat Ventricular Arrhythmia Catheter Ablation: A Case Report

**DOI:** 10.7759/cureus.100206

**Published:** 2025-12-27

**Authors:** Roxana Farokhnia, Aakshi Sanchorawala, Udaya B Padakandla, Praveen Rao, Saravanan Ramamoorthy

**Affiliations:** 1 College of Medicine, Texas A&M College of Medicine, Dallas, USA; 2 Anesthesiology and Perioperative Medicine, US Anesthesia Partners, Dallas, USA; 3 Anesthesiology, Baylor University Medical Center, Dallas, USA; 4 Cardiology, Baylor Scott &amp; White Cardiology Consultants of Texas, Dallas, USA

**Keywords:** catheter ablation, premature ventricular complex, regional anesthesia, ventricular arrhythmia, ventricular tachycardia

## Abstract

Catheter ablation procedures for ventricular arrhythmias are often performed under general anesthesia or total intravenous anesthesia to prevent patient movement and discomfort. However, anesthetic agents such as propofol, opioids, and dexmedetomidine can suppress ventricular arrhythmias, preventing successful mapping and ablation during the procedure. Here, we present the case of a 25-year-old female with premature ventricular contractions (PVC) and ventricular tachycardia (VT) who underwent a successful catheter ablation procedure using regional anesthesia after two previously unsuccessful ablation attempts. The first attempt used total intravenous anesthesia to perform the ablation. However, patient movement complicated the mapping, and ventricular arrhythmias returned in a few weeks. The second procedure used decreased total intravenous sedation to reduce arrhythmia suppression; however, no arrhythmias were able to be induced, and the patient continued to have episodes of ventricular tachycardia. To prevent potential arrhythmia suppression from the prior anesthetic agents used, a different anesthetic approach was used for the third attempt: ultrasound-guided peripheral nerve blocks of the right ilioinguinal, iliohypogastric, obturator, and lateral femoral cutaneous nerves. To the authors’ current knowledge, there have been no reports in the literature describing regional anesthesia as the primary anesthetic technique specifically to avoid arrhythmia suppression during ventricular arrhythmia catheter ablation procedures. This technique enabled successful arrhythmia inducibility, which in turn allowed for accurate mapping and ablation.

## Introduction

Catheter ablation procedures for ventricular arrhythmias have become more common due to advancing technology and high success rates. However, the anesthetic approach to these procedures remains complex and requires further exploration [1]. General anesthesia is most commonly used for catheter ablation procedures to prevent patient movement and discomfort; however, the use of minimal sedation and local anesthesia has also been studied for ventricular arrhythmia ablation [2]. Important consideration regarding anesthetic agents such as propofol, opioids, and dexmedetomidine must be taken, as evidence suggests these medications can suppress ventricular arrhythmias and impede effective mapping and ablation [2-5]. The potential antiarrhythmic mechanisms of propofol include ion channel inhibition, uneven suppression of the autonomic nervous system, and protection of gap junctions during ischemia [6]. Opioids can inhibit sympathetic reflexes, which can suppress VT induction [2]. Dexmedetomidine's anti-arrhythmogenic mechanism includes increasing vagal activity that prolongs repolarization and refractory periods, which decreases cardiac automaticity; binding to alpha-2 receptors, which creates a negative feedback loop to reduce the release of catecholamines, which are a mediator of ventricular arrhythmias; and potentially inhibiting calcium and sodium channels on myocytes directly [7]. These mechanisms collectively may result in incomplete or absent arrhythmia mapping, which can, in turn, lead to persistent ventricular arrhythmias associated with significant morbidity and mortality.

This report highlights a case in which the use of total intravenous anesthesia in a catheter ablation procedure resulted in return of ventricular arrhythmias, requiring a second procedure with lighter sedation that was not able to induce any ventricular arrhythmias. As a result, a novel regional anesthesia technique targeting the right ilioinguinal, iliohypogastric, obturator, and lateral femoral cutaneous nerves under ultrasound guidance was used. To the authors' current knowledge, there have been no reports in the literature regarding the use of regional anesthesia instead of total intravenous anesthesia for the primary anesthetic technique and prevention of arrhythmia suppression during cardiac ablation procedures for ventricular arrhythmias.

## Case presentation

A 25-year-old female with paroxysmal atrial fibrillation (AF) and premature ventricular contractions (PVC) presented for a repeat right ventricular outflow tract (RVOT) ablation due to ventricular arrhythmias refractory to medication management.

The patient was found to have PVCs and likely idiopathic ventricular tachycardia (VT) with symptoms of palpitations. Despite treatment with metoprolol succinate 25 mg, she had breakthrough episodes. The patient opted for an ablation procedure to prevent lifelong medication management of the condition. For the first procedure, the patient was induced intravenously with 20 mg of lidocaine, 50 mcg of fentanyl, and 280 mg of propofol bolus. Throughout the procedure, the patient received 790.94 mg propofol infusion, 32 mcg of isoproterenol, and 750 mcg of phenylephrine. Ablation of the PVCs and VT arising from the RVOT was technically challenging because patient movement, likely due to discomfort despite sedation, interfering with accurate mapping of the focus origin of the arrhythmia. Even with these difficulties, acute procedural success was ultimately achieved. Two weeks after the procedure, the patient developed significant chest pain and was diagnosed with pericarditis, which was treated with colchicine and ibuprofen. She continued the colchicine 0.6 mg twice daily and one week later went to the emergency department for a heart rate >200. She was found to have AF with rapid ventricular response (RVR). Holter monitoring confirmed recurrent symptomatic VT, in addition to episodes of AF with RVR that was likely secondary to her post-ablation pericarditis. She was started back on metoprolol succinate 25 mg once daily. Despite the medication, she continued to have tachycardia with angina, which led to another emergency department visit where they added flecainide 100 mg twice daily and increased metoprolol succinate to 50 mg once daily. The patient opted for a repeat ablation procedure.

**Figure 1:**
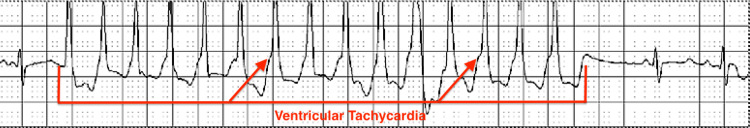
Electrocardiogram from Holter monitor demonstrating VT. VT: ventricular tachycardia.

For the second ablation procedure, the patient was intravenously induced with decreased total intravenous sedation compared to her first ablation procedure to attempt to identify the focus of the ventricular arrhythmia. Given the suspected role of propofol and fentanyl in suppressing arrhythmias during the first procedure, the dose of propofol was reduced, fentanyl was removed, and dexmedetomidine was added to reduce patient movement. The patient received a 160 mg propofol bolus, 162.84 mg propofol infusion, and 12.7 mcg of dexmedetomidine. During the procedure, neither supraventricular tachycardia (SVT) nor VT could be induced with a 2 mg adenosine injection, 15 mcg of isoproterenol, or burst pacing. The patient was woken up completely, and an arrhythmia was still not able to be induced. The patient had been off of flecainide and metoprolol for two weeks before the procedure, and it was thought that her tachycardia could have been a result of residual symptoms from her pericarditis. No ablation was performed. Four months after the second procedure, another Holter monitor still showed recurrent symptomatic VT, up to 230 bpm. Over the next three months, the patient had recurrent intermittent palpitations with a repeat monitor showing recurrent short runs of nonsustained VT (Figure 1). She was placed on diltiazem and flecainide with control of symptoms but opted to attempt another ablation procedure.

For the third procedure, a regional anesthesia approach was used to minimize movement and provide adequate pain control without any risk of arrhythmia suppression from anesthetic agents such as propofol, fentanyl, or dexmedetomidine. We believed that this technique would provide more extensive dermatomal coverage and deeper analgesia than local anesthesia alone, thereby reducing movement and anxiety without affecting arrhythmia inducibility. Ultrasound-guided peripheral nerve blocks of the right ilioinguinal, iliohypogastric, obturator, and lateral femoral cutaneous nerves were performed by administering 10 mL of 1.5% mepivacaine and 30 mL of 0.5% ropivacaine with aspiration every 5 mL. Patient weight was 56.6 kg; therefore, the patient was well under the threshold for the maximum recommended ropivacaine dose (3 mg/kg without epinephrine, max dose 169.8 mg) or mepivacaine dose (7 mg/kg without epinephrine, max dose 396.2 mg) to prevent the potential risk of cardiotoxicity at high doses. We used a linear probe with a frequency of 2.5-12 MHz for 2D plane using an in-plane technique with the patient in the supine position. For the ilioinguinal and iliohypogastric nerve block, the linear probe was placed transversely along the line from the anterior superior iliac spine (ASIS) to the umbilicus with the superior portion of the transducer facing the umbilicus. The superior portion of the transducer was slowly rotated superiorly and inferiorly until the internal oblique and transversus abdominis muscles were identified. The iliohypogastric nerve lies medially to the ilioinguinal nerve in this plane. The needle tip was placed at the lower border of the ultrasound inducer and was introduced between the internal oblique and transverse abdominis muscle layers in the fascial plane. A small amount of normal saline was injected to separate the layers of the muscle. The fascial sheaths of the internal oblique and transversus abdominis muscles were identified, and 10 mL of anesthetic was injected for each nerve. For the lateral femoral cutaneous nerve block, the linear probe was placed transversely just inferior and medial to the ASIS. From lateral to medial, the needle tip targeted the fascial plane between the sartorius and iliacus muscles. 10 mL of anesthetic was deposited deep to the fascia iliaca, just medial to the ASIS. For the obturator nerve block, the linear probe was placed transversely over the medial aspect of the proximal thigh at the inguinal crease. From lateral to medial, the needle tip with 10 mL of anesthetic is injected between the adductor longus and adductor brevis muscles to target the anterior branch. Sensory blockade was confirmed via pinprick testing using a clamp. Minimal motor blockade was observed, primarily in the obturator nerve distribution, consistent with the limited motor involvement expected from this block combination.

These blocks provided satisfactory coverage for the T12-L4 dermatomes at the level of the inguinal ligament and right groin throughout the procedure. Close monitoring of blood pressure, heart rate, and ECG was performed throughout the procedure as well as monitoring for adequate pain control. A micropuncture needle was used to gain femoral access. The right femoral artery had a 4Fr sheath placed for invasive hemodynamic monitoring. The right femoral vein had 7Fr, 7Fr, 8Fr, and 9Fr sheaths placed. An inquiry decapolar catheter was placed into the coronary sinus for left atrial stimulation and recording. The patient remained comfortable with no movement, classifying as a 0 on the Richmond Agitation-Sedation scale. Successful mapping was achieved. Administration of isoproterenol stimulated PVCs and runs of nonsustained VTs. Throughout the ablation procedure, the patient received 10,000 units of heparin, 1000 mg of acetaminophen, 8 mg of dexamethasone, 50 mg of diphenhydramine, and 32 mcg of isoproterenol. She also received 150 mcg of fentanyl after the arrhythmia was induced and improved mapping was achieved, in place of midazolam, which could potentially suppress arrhythmias. This was given to address her chest discomfort and anxiety during the ablation. Successful ablation of PVCs at the RVOT was achieved with no further PVCs noted after repeat testing with isoproterenol.

## Discussion

Ultrasound-guided peripheral nerve blocks of the dermatomal regions of the ilioinguinal (L1), iliohypogastric (T12-L1), obturator (L2-L4), and lateral femoral cutaneous nerves (L2-L3) are a reasonable option for providing targeted pain relief in patients with PVCs undergoing VT ablation [8]. This block combination provides relief of the lower abdomen, medial thigh, lateral thigh, groin, and upper hip, facilitating femoral access throughout the duration of catheter ablation without the use of sedatives that may suppress arrythmias. In addition, this block combination provides greater dermatomal coverage than individual blocks such as fascia iliaca (L2-L4) and lumbar plexus blocks (L1-L4) [9]. Peripheral nerve blocks are generally associated with minimal systemic effects, and safety considerations for this anesthetic technique are the same as any traditional regional nerve block and include damage to the nerve, bleeding, local anesthetic systemic toxicity, allergic reactions, and infection [10]. Close monitoring of blood pressure, heart rate, and ECG is essential throughout the procedure as well as monitoring for adequate pain control. However, effective nerve blocks can also allow for earlier postoperative ambulation; decreased postoperative risks associated with immobility, such as deep vein thrombosis; and decreased hidden costs of procedures, including length of hospitalization [9,11,12].

Previous studies comparing local anesthesia with minimal sedation to general anesthesia during cardiac ablation in patients with PVC showed that local anesthesia with minimal sedation is comparable to general anesthesia. General anesthesia is more likely to cause complications of PVC inhibition and PVC disinhibition post-extubation [13]. However, local anesthesia with minimal sedation may provide insufficient dermatomal or deep tissue coverage for the degree and duration of stimulation required during ablation, making peripheral nerve blocks a more robust alternative in select cases [14]. In addition, spinal anesthesia has also been used in some cases of catheter ablation. A 2024 case report by Jain et al. used spinal anesthesia as the anesthetic for a successful ablation procedure for refractory left-sided VT. However, the authors do note a limitation that spinal anesthesia would not be appropriate for longer ablations because there is limited durability before recurrence of pain [15]. In our case, spinal anesthesia was contraindicated due to the planned use of intraoperative heparin, which requires a delay of at least two hours post-neuraxial block placement. Furthermore, given the patient's anxiety and possibility of distress from bilateral lower extremity motor blockade, spinal anesthesia was considered an unsuitable option. Another anesthetic strategy to prevent ventricular arrhythmia suppression would be to use volatile anesthetics to avoid propofol, opioids, or dexmedetomidine. This technique has important safety considerations as it can prolong the QT interval and increased risk of hemodynamic instability requiring increased vigilance [16,17].

There were some limitations to the case report. First, due to our patient's young age and otherwise lack of co-morbidities, generalization of this regional anesthesia approach to other populations, such as the elderly and those with multiple co-morbidities, is limited. Additionally, the success of a regional anesthesia nerve block technique can partially be attributed to the skill and experience of the operator, which can cause varying results. Logistical factors such as procedure duration tolerance, block onset time, need for supplemental analgesia, variability in sympathetic blockade from potential hemodynamic effects of nerve blocks, and resource requirements must also be considered for application of this technique to other cases. Our patient was also able to tolerate the procedure without any sedation for anxiety while mapping and ablating her ventricular arrhythmias during the third ablation procedure. However, for patients with greater anxiety, this approach might not be the best option. Further studies and reports that explore the use of regional anesthesia for catheter ablation in different populations and different regional techniques should be considered.

Administering regional anesthesia is a reasonable potential alternative strategy requiring further validation for cardiac catheter ablation procedures for ventricular arrhythmias. This case report provides an opportunity to showcase the use of peripheral nerve blocks to provide anesthesia during VT and PVC ablations. General anesthesia is often first-line for ventricular arrhythmia ablation to minimize patient movement and optimize patient comfort; however, as seen in this case, when unsuccessful, it can result in multiple procedures and patient discomfort [2,3]. Regional anesthesia can provide adequate pain control, reduced movement, and preserved arrhythmia inducibility, thereby enabling successful mapping and ventricular arrhythmia catheter ablation.

## Conclusions

Catheter ablation procedures for ventricular arrhythmias are often performed under general anesthesia. However, anesthetic agents can suppress ventricular arrhythmias, which can prevent accurate mapping and ablation. This case report showcases the use of peripheral nerve blocks to provide anesthesia during VT and PVC ablations. Although our study is limited by a single-case design, future cases may consider regional anesthesia as an option when ventricular arrhythmias are not able to be induced under general anesthesia. Further research is needed to assess the safety, feasibility, and reproducibility of this approach before recommending routine use of regional anesthesia for ventricular arrhythmia ablation procedures.
